# Comparison of different internal fixation models in ankle arthrodesis using 3D finite-element analysis

**DOI:** 10.1186/s40001-023-01554-0

**Published:** 2023-12-08

**Authors:** Bo Feng, Qing-bo Gao, Guang-ming Dai, Ke-cheng Niu, Wei Jiang, Zhen-yu Wang, Hao-yan Zheng

**Affiliations:** Department of Orthopaedic Medicine, Third Affiliated Hospital of Inner Mongonia Medical University, No.20 of Shaoxian Road, Kundulun District, Baotou, 014010 China

**Keywords:** Ankle arthrodesis, Biomechanics, Digital orthopedics, Internal fixation, 3D finite element

## Abstract

**Background:**

The purpose of this study is to use three-dimensional finite-element analysis to better understand the biomechanical features of various internal fixators for ankle arthrodesis.

**Methods:**

We used finite-element analysis to compare four different types of internal fixations in ankle arthrodesis: Group A had three crossed screws (Ø6.5 mm); Group B had two crossed screws (Ø6.5 mm) and an anterior plate (Ø2.7 mm); Group C only had an anterior anatomical plate (Ø3.5 mm); Group D had one anterior anatomical plate (Ø3.5 mm) and one posterior–lateral screw (Ø6.5 mm). We adopted Ansys 21.0 software to analyze and compare the four types in terms of the displacement of the arthrodesis surface and the stress peak and stress distribution of these models under intorsion, extorsion, dorsiflexion torque, and neutral vertical load.

**Results:**

① Displacement of the arthrodesis surface: In Group A, the maximum displacement was larger than Group D under neutral vertical load and dorsiflexion torque but less than it under intorsion and extorsion torque. In Group B, the maximum displacement against dorsiflexion, neutral vertical load, intorsion, and extorsion was less than that in the other three fixation models. In Group C, the maximum displacement against the above four loading patterns were significantly higher than that in another three fixation models. ② Stress peak and stress distribution: based on the stress distribution of the four models, the peak von Mises stress was concentrated in the central sections of the compression screws, plate joints, and bending parts of the plates.

**Conclusions:**

The fixation model consisting of two crossed screws and an anterior outperformed the other three fixation models in terms of biomedical advantages; thus, this model can be deemed a safe and reliable internal fixation approach for ankle arthrodesis.

## Background

At the very moment, there is no clear gold standard for the treatment of end-stage ankle osteoarthritis—but fusion surgery still plays an important role. Ankle arthrodesis is currently recognized as a good choice due to its higher healing rate and a wider range of indications for use [1]. Ankle arthrodesis was first proposed by Austrian surgeon Eduard Alber in 1879, and since then, dozens of surgical approaches have been developed by through continuous refinements of the technique [2]. The effectiveness of ankle arthrodesis is ensured by an adequate and effective contact area, as well as a strong and stable fixation and compression. It can alleviate discomfort, rectify deformities, realign lower limbs, and help reconstruct plantigrade feet [2, 3]. The selection of internal fixators is particularly important for ankle arthrodesis, and there are numerous fixation methods available, including compression screw internal fixation, intramedullary nail fixation, external support fixation, and internal plate fixation; however, there is still a non-healing rate of 5–37% [4]. Different internal fixators have different biomechanical properties, and there is no consensus on the optimal placement position and orientation. Therefore, we achieved satisfactory efficacy in this study by performing ankle arthrodesis with two crossed screws (Ø6.5 mm) and anterior plates (Ø2.7 mm). Then, we compared this with other commonly used internal fixation methods using the biomechanical finite-element analysis to explore the biomechanical characteristics of different internal fixators and provide a better solution for optimizing the fixation of ankle arthrodesis, thereby laying a theoretical foundation for future clinical research.

## Materials and methods

### Study participants

We selected a healthy male adult, 175 cm in height and weighing 65 kg, as the volunteer (with no history of any foot and ankle disease, trauma, or other relevant conditions), who signed the informed consent form. This study was approved by the Ethics Committee of Inner Mongolia Baogang Hospital.

### Study method

#### Preliminary creation of a 3D ankle model

We performed a 64-slice spiral CT plain scan of the right ankle of the volunteer. We first isolated and retrieved the bone tissue structure of the ankle by importing the collected CT image data in the DICOM format into the Mimics software. We then used denoising and segmentation to create a clear and accurate preliminary 3D tibiotalar model. We imported the relevant files into Geomagic 2017 software to align the resulting tibiotalar model with the actual circumstances. We created a 3D tibiotalar model by replicating the process of joint cleaning for ankle arthrodesis. We removed the distal tibia as well as cartilage and subchondral tissue from the astragalar dome.

#### Creation of 3D models of the four internal fixation types for ankle arthrodesis

**Fig. 1:**
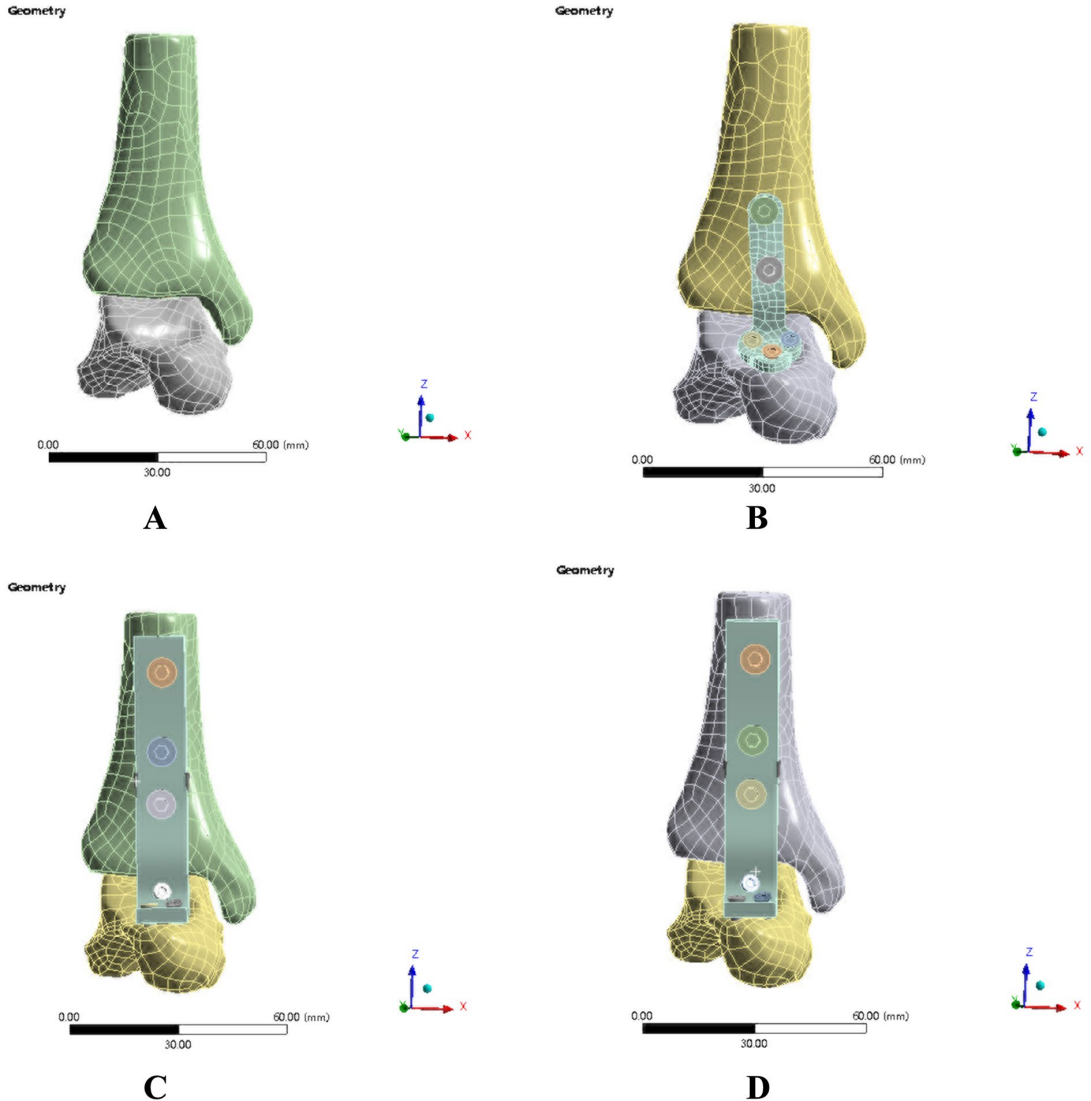
Schematic diagram of appearance of three-dimensional model of ankle arthrodesis with four internal fixation modes

**Fig. 2:**
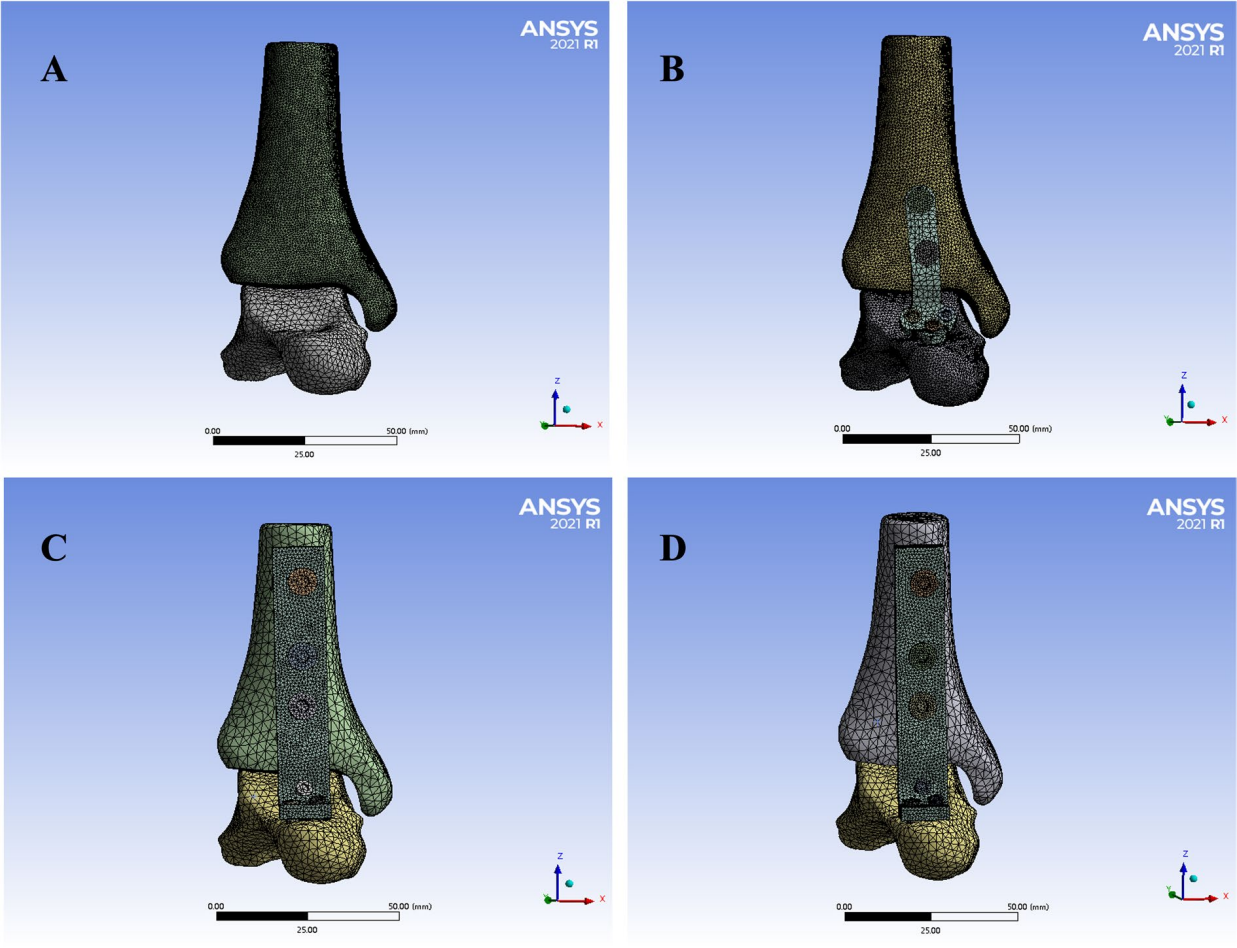
Meshing the four internal fixation modes of ankle arthrodesis three-dimensional model

We used the interference steps in Solidworks 2017 software to replicate the procedure of ankle arthrodesis utilizing four distinct internal fixation models. Finally, we conducted a finite-element analysis of the four fixation models, as shown in Figs. 1, 2 and Table. 1. We converted bone apparent density [*ρ* (kg m^−3^)] to Young’s modulus [*E* (MPa)]. *E* = 0:2*ρ*^1:52^ (for *ρ* ≤ 476:7) and *E* = − 3842 + 13*ρ* (for *ρ* > 476:7) based on earlier research [5, 6]. Neither sliding nor separation was permitted between the contact surfaces. The screw has a Young’s modulus of 200 GPa. For the bones and screws, a Poisson’s ratio of 0.3 was assumed. Frictionless contacts are characterized as bones and screws. Kuhn surfaces were utilized on all outside surfaces to improve contact normal calculation accuracy.

**Table 1:** Information of the mesh structure created for 3D finite-element analysis

Group	Nodes	Units
Group 1	1,053,770	740,650
Group 2	227,665	130,803
Group 3	80,818	46,156
Group 4	111,165	61,725

#### Determination of contact boundary conditions and loads

**Fig. 3:**
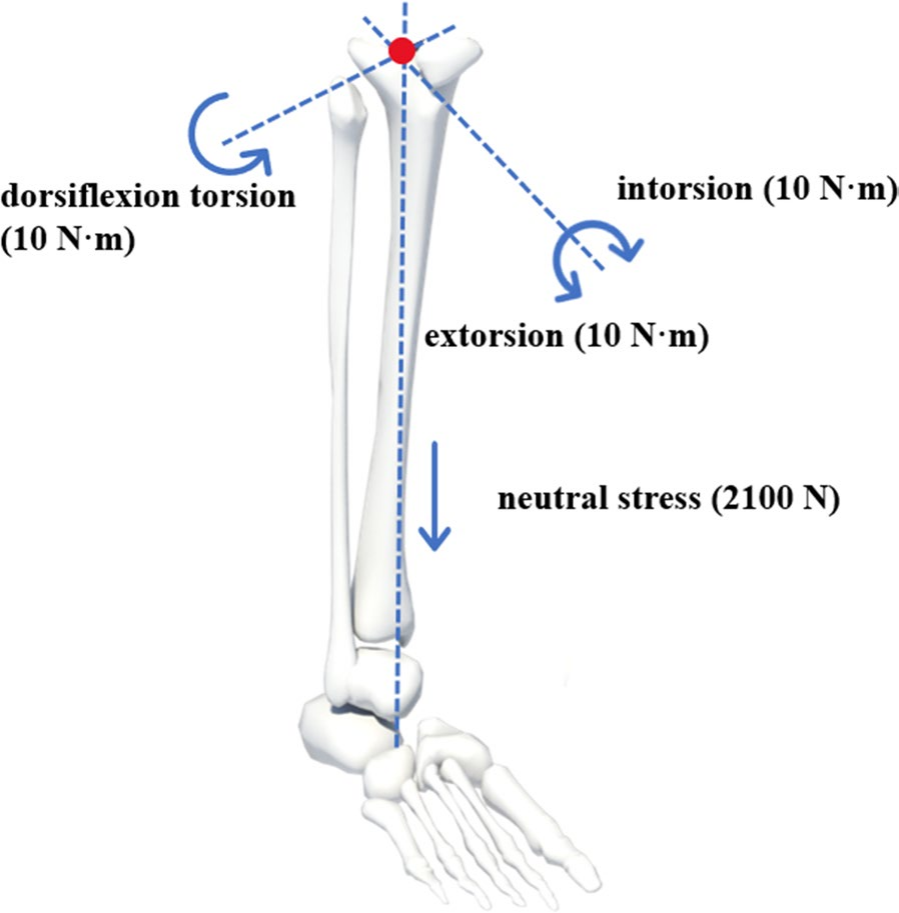
Applied intorsion torque (10 N m), extorsion torque (10 N m), dorsiflexion torque (10 N m), and neutral vertical load (2100 N) on the upper surface of the tibia for each model

**Fig. 4:**
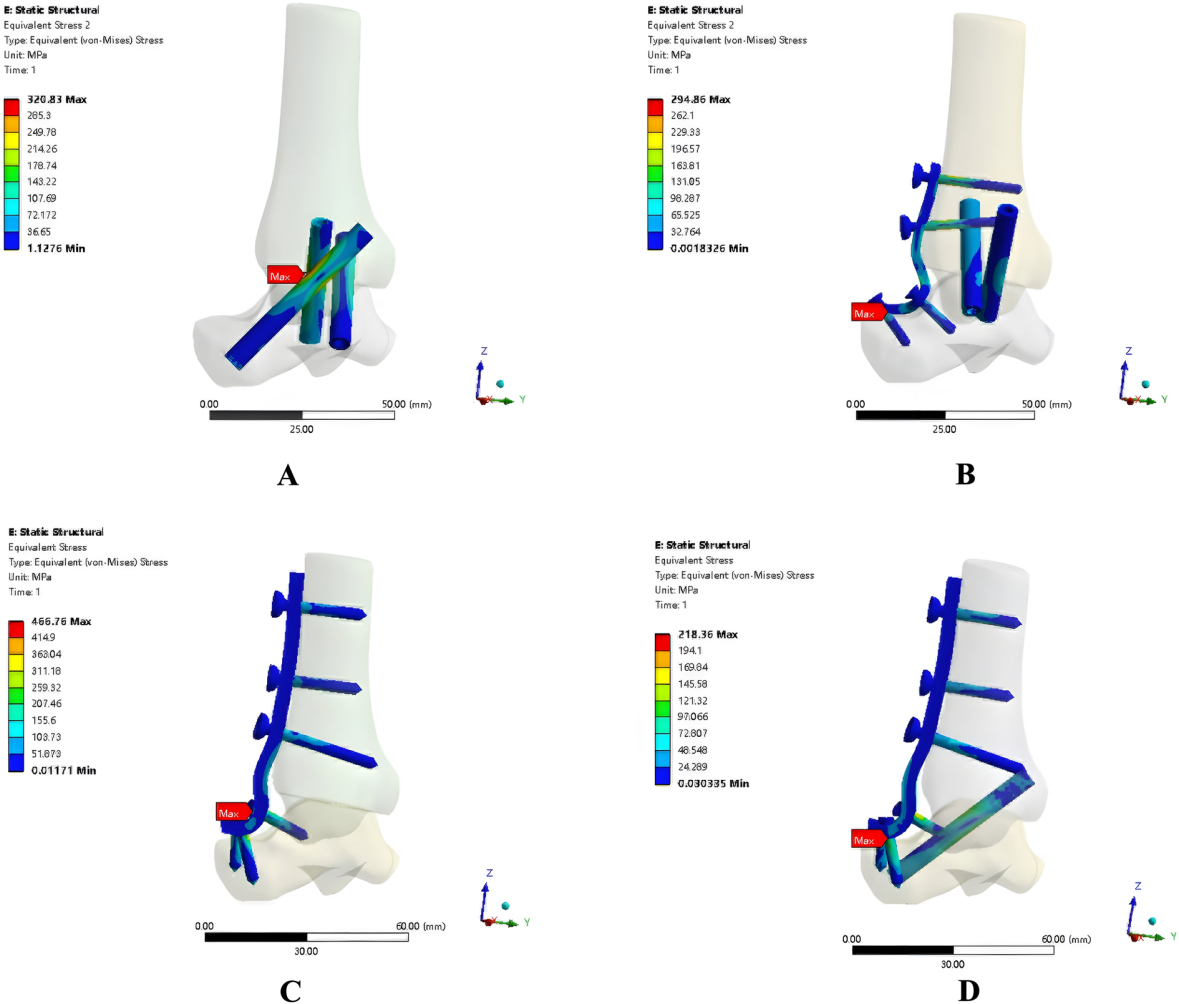
The von Mises stress distribution of the four different fixation models

**Fig. 5:**
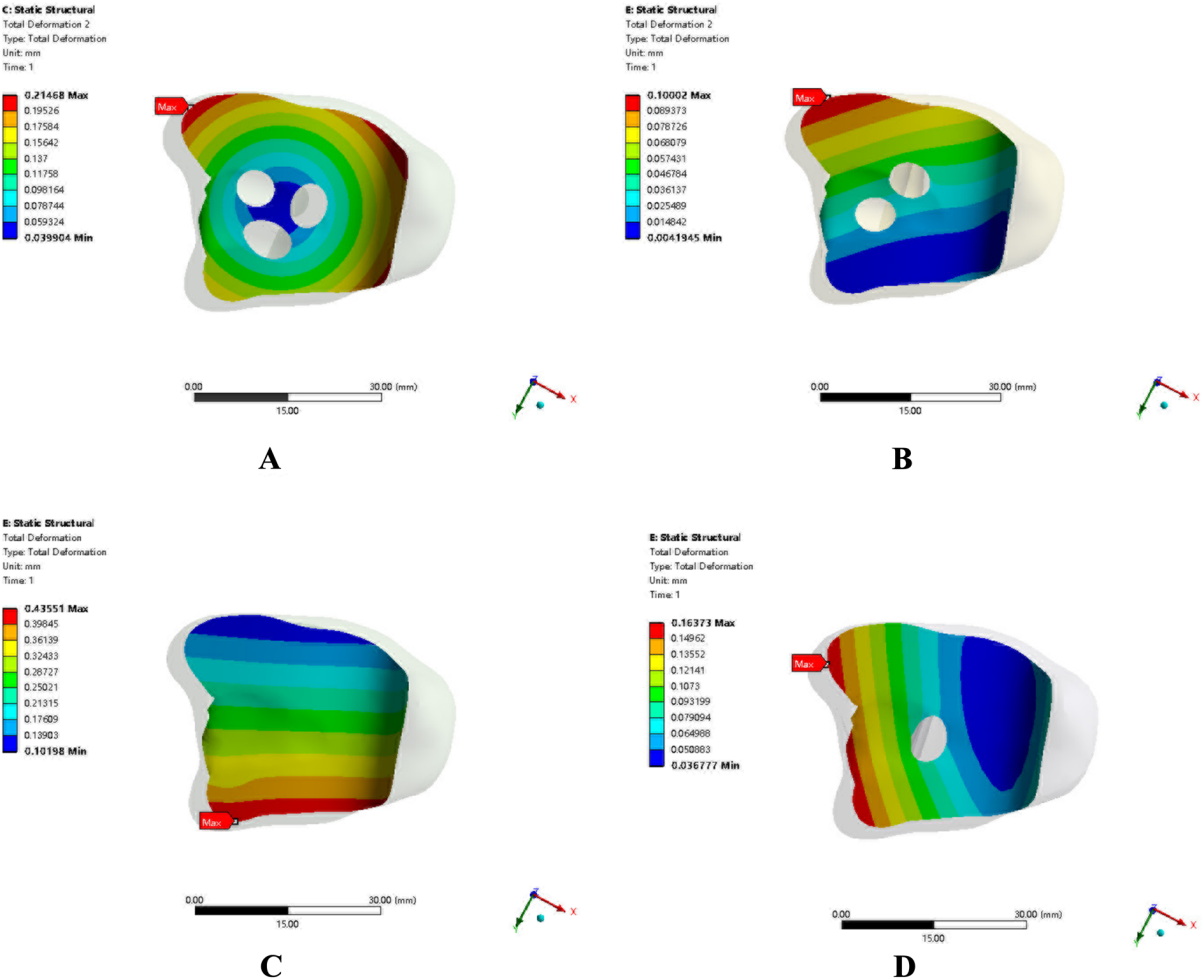
The displacement cloud atlas of the four different fixation models

Referring to our previous experimental study [7, 8], and based on the actual motion of the ankle joints while walking, we fixed the lower part of the astragalus, and simulated the mechanical changes of ankle joints under dorsiflexion, neutral, internal rotation and external rotation stresses. Then, as per our earlier research [5, 6, 9], we applied intorsion torque (10 N m), extorsion torque (10 N m), dorsiflexion torque (10 N m), and neutral vertical load (2100 N) on the upper surface of the tibia for each model, as shown in Fig. 3. The von Mises stress distribution and displacement cloud atlas of the four different fixation models are depicted in Figs. 4 and 5, respectively.

#### Assessment indicators

We primarily evaluated the stability and safety of the internal fixators. Specifically, we assessed stability by observing and analyzing the maximum displacement of the ankle arthrodesis surface and assessed the safety by observing the stress distribution and stress peak of the plates, screws, and bone. Plastic materials, such as stainless steel, often experience yield failures. We used the von Mises stress as a failure criterion based on the fourth strength theory. Thus, we used von Mises stress to assess the stress state of steel plates, screws, and bones.

## Results

### Displacement of the arthrodesis surface

Table 2 displays the maximum displacement of the arthrodesis surface for each of the four ankle arthrodesis models subjected to four stressors. The model with two crossing screws and one anterior plate had the smallest maximum displacement under dorsiflexion torsion, extorsion, intorsion, and neutral vertical load when compared to the other three fixation models. The maximum displacement against the aforementioned four loading patterns was noticeably larger in the fixation model with anterior plates alone compared to the other three fixation models. The maximum displacement in the model with three screws was larger than in the model with anterior plates and posterior–lateral screws under neutral vertical load and dorsiflexion torque. The maximal displacement of the arthrodesis surface under intorsion and extorsion torque was less in the fixation model with three screws compared to the one with anterior plates and posterior–lateral screws.

**Table 2:** Maximum displacement of the fusion surface (mm)

Fixation type	Neutral	Dorsiflexion	Extorsion	Intorsion
Fixation with three screws	0.196	0.194	0.209	0.215
Fixation with two crossed screws and anterior plate	0.100	0.059	0.139	0.135
Fixation with anterior plate	0.436	0.373	1.369	1.316
Fixation with anterior plate and posterior–lateral screws	0.164	0.068	0.251	0.246

### Stress peak and stress distribution

Tables 3, 4, 5, 6, 7 display the stress distribution and stress peaks for the four different fixation models. When we compared the overall stress peaks of the four models, we found that with respect to extorsion and intorsion torque, the stress peaks in the fixation model using two crossed screws and anterior plates and in the fixation model with three screws were smaller than that in the fixation model with anterior plates and the fixation model with anterior plates and posterior–lateral screws. As for neutral vertical load and dorsiflexion torque, the stress peak in the fixation model with two crossed screws and anterior plates and the fixation model with anterior plates and posterior–lateral screws was smaller than that in the fixation model with three screws and the fixation model with anterior plates. The fixation model with anterior plates alone was the model with the highest stress peak across all four forces. In terms of the stress peaks of bones, plates, and screws in the four models, the fixation model with two crossed screws and anterior plates and that with three screws had the highest probability of fractured screws and plates and stress fracture under vertical load. The fixation model with anterior plates alone and that with anterior plates and posterior–lateral screws had the highest probability of fractured screws and plates and stress fracture under intorsion and extorsion stress pattern. Based on the stress maps of all four models, it is clear that the compression screws, plate joints, and bending elements of the plates are all experiencing significant amounts of stress in their central regions.

**Table 3:** Overall stress peak of the four models (MPa)

Fixation type	Neutral	Dorsiflexion	Extorsion	Intorsion
Fixation with three screws	320.83	210.46	154.99	200.04
Fixation with two crossed screws and anterior plate	294.86	130.97	175.13	154.66
Fixation with anterior plate	466.76	320.36	637.44	661.89
Fixation with anterior plate and posterior–lateral screws	218.36	155.43	256.95	234.48

**Table 4:** Stress peak of the model with three screws (MPa)

Materials	Neutral	Dorsiflexion	Extorsion	Intorsion
Stress peak of screws	320.83	210.46	154.99	200.04
Stress peak of bones	69.57	43.495	53.237	56.135

**Table 5:** Stress peak of the fixation model with two crossed screws and anterior plate (MPa)

Materials	Neutral	Dorsiflexion	Extorsion	Intorsion
Stress peak of plates	294.86	123.77	132.74	137.07
Stress peak of screws	270.57	130.97	175.13	154.66
Stress peak of bones	171.13	76.952	82.092	58.289

**Table 6:** Stress peak of the model with anterior plate (MPa)

Materials	Neutral	Dorsiflexion	Extorsion	Intorsion
Stress peak of plates	466.76	301.73	587.59	619.35
Stress peak of screws	428.16	320.36	637.44	661.89
Stress peak of bones	83.613	63.013	104.13	75.594

**Table 7:** Stress peak of the model with anterior plate and posterior–lateral screws (MPa)

Materials	Neutral	Dorsiflexion	Extorsion	Intorsion
Stress peak of plates	113.27	103.75	129.23	140.26
Stress peak of screws	218.36	155.43	256.95	234.48
Stress peak of bones	72.635	64.396	78.228	79.857

## Discussion

The human body’s ability to bear weight and move forward depends on the proper functioning of the ankle joint. Ankle arthroplasty and ankle arthrodesis are two surgical procedures used to address paralytic deformity with muscle–tendon imbalance and end-stage joint diseases such post-traumatic arthritis of the ankle, chronic ankle instability, rheumatoid arthritis, tuberculous or suppurative ankle arthritis, and ischemic necrosis of the astragalus [10]. Recent years have seen significant advancements in ankle arthroplasty techniques; nevertheless, these have seen limited clinical application because of narrow indications, increased technical requirements, and problems including infection and loosening of prostheses [11, 12]. Ankle arthrodesis is a surgical procedure that realigns the bones around a joint to treat lesions, alleviate pain, restore proper alignment, strengthen the joint, and enhance its function. The compression effect must be powerful and dependable, and there must be sufficient contact area for it to work. Careful consideration must be given when choosing internal fixators. There have been numerous studies comparing the biomechanical and clinical efficacy of different internal fixation techniques for ankle arthrodesis [13–15]. Studies have found that fixation with screws had greater advantages in terms of the fusion rate and complication control, especially after the development and widespread use of fixation with three compression screws [8, 16, 17]. In arthroscopic fusion, most of the capsule is preserved, potentially adding to the stability of the fixation. Nonetheless arthroscopic fusion allows only for screw fixation.

Ankle arthrodesis has evolved in recent years to include the use of multiple locking plate types and orientations. The anterior plates have been shown through biomechanical testing to function as “tension bands” that efficiently resist ankle flexion and extension, hence minimizing micro-motion of the ankle arthrodesis surface. They can also aid in bone repair and greatly increase rotational resistance [18–20]. The ankle joint can be more thoroughly exposed, and a clear surgical field can facilitate the procedure. Although, based on the two crossed screws, the third posterior–lateral screw can significantly resist dorsiflexion and rotational stresses and increase the initial stability; its placement can easily damage the peroneal nerve and cause the screws to collide with each other. With the aid of image analysis software, So et al. [21] compared and analyzed the proportion of loss of articular surface area (SA) on the top of the astragalus between the traditional fixation with two screws and that with three screws. The results showed that the third posterior–lateral screw created an oval hole due to the small entry angle and non-perpendicular direction, which resulted in a loss of 6%–10% of the subtarsal joint SA; therefore, cautious use of this screw is required in patients with low bone mass. So et al. proposed using locking plates in combination with minimal screw fixation. We have been performing ankle arthrodesis using the fixation with two crossed screws (Ø6.5 mm) and anterior plates (Ø2.7 mm) through a small anterior incision since 2020, after carefully considering the benefits and drawbacks of various internal fixation models and the choice of access. The biomechanical features could not be identified, although there were satisfactory therapeutic results.

Finite-element analysis is a dynamic, widely utilized, practical, and efficient numerical analytical method that has swiftly extended from structural engineering strength analysis to practically all sectors of science and industry thanks to the rapid advancement and popularity of computer technology. In 1972, it was initially used in orthopedic biomechanics to measure bone stresses [22]. Since then, it has been increasingly applied in stress analysis of bones and bone prosthesis, fracture fixation devices, and non-bone tissues to assess the relationship between load-bearing function and morphology, and provide a theoretical basis for clinical practice, thus optimizing techniques for implant design and fixation. Finite-element analysis can simulate working conditions that cannot be produced in conventional biomechanical experiments and can be used for static or dynamic analytic research, with the advantages of short duration, low cost, repeatability, and comprehensive performance testing. Additionally, it can complement conventional biomechanical experiments, and provide more comprehensive, accurate, three-dimensional, and diversified mechanical data for clinical practice. In recent years, the finite-element method has been increasingly used in the study of biomechanics of ankle joints [23–26]. Finite-element analysis has been used in the analysis of ankle arthrodesis. Wang et al.'s study focused on the initial stability of three-screw fixation for ankle arthroscopic anthrosis. The screw configuration of the posteromedial home-run screw was found to avoid collisions and was biomechanically more stable than that of the posteromedial home-run screw [27]. Zhu et al. discussed initial stability and stress distribution of ankle arthroscopic arthrodesis with three kinds of two-screw configuration fixation [28]. However, there is no finite-element analysis focusing on the mechanical properties of the anterior plate in ankle arthrodesis for the time being. In addition, a cross-sectional comparison of the mechanical properties of multiple ankle arthrodesis internal fixation modalities by finite-element analysis is urgently needed to provide reliable evidence support for further clinical studies and clinical practice.

In this study, we collected the CT scan image data of a healthy adult male volunteer and imported this into the Mimics software. Based on this, we created a rough 3D ankle joint model. We performed smoothing and denoising of the model using the Geomagic software, to assemble a solid model. Then, we imported the data into the Solidworks software for assembly and cutting, and simulating procedures such as ankle arthrodesis. With this, we created the experimental models for four types of ankle arthrodesis, for finite-element analysis. Finally, we imported the models into the finite-element analysis software Ansys, and set the analysis parameters and properties, as well as the loading conditions, to obtain the final analysis results. The 3D finite-element model established in this study can fully obtain spatial information from different angles, and the design of various parameters approximates clinical reality; therefore, this study has a high degree of simulation and validity.

The microdisplacement of the arthrodesis surface refers to the deformation of the arthrodesis surface of the ankle under the intorsion, extorsion, dorsiflexion torsion, and neutral stresses based on the gait during walking. The displacement value can indicate the effectiveness and stability of internal fixators for ankle arthrodesis. The results of this study revealed that in the fixation model with anterior plates alone, the displacement of the arthrodesis surface under neutral and dorsiflexion torsion was significantly smaller than that under extorsion and intorsion, indicating that anterior plates had significant advantages against neutral and dorsiflexion torsion; however, it was slightly weak in resisting intorsion and extorsion. In the model with a 6.5 mm posterior–lateral screw based on anterior plates, the displacement under neutral stress, dorsiflexion intorsion, extorsion, and intorsion was significantly smaller than that in the fixation model with anterior plates, specifically under intorsion and extorsion, indicating that the placement of the posterior–lateral screw can produce a better fixation effect. The findings of Clifford [29] and Xie [30] also confirm this theory. As for the fixation model with three screws, there was a small difference in the degree of deformation of the arthrodesis surface under the four forces, indicating that this model can provide more balanced resistance to external forces. The fixation model with three screws was more advantageous than the fixation model with anterior plates and posterior–lateral screws in resisting intorsion and extorsion; the anterior plates performed satisfactorily in resisting neutral stress and dorsiflexion intorsion, specifically the dorsiflexion intorsion. The fixation model with anterior plates and posterior–lateral screws performed better in terms of displacement of the arthrodesis surface.

Our analysis of the experimental results revealed that the fixation model with two crossed screws and anterior plates was undoubtedly the most stable configuration among the four models, and the displacement of the arthrodesis surface against neutral stress, dorsiflexion intorsion, intorsion, and extorsion was smaller than that in the other three models. Based on the stress distribution and stress peak, most forces were concentrated in the central sections of the compression screws, plate joints and bending parts of the plates. This indicates that the components used in these parts should be thickened and reinforced, to prevent fractured screws and plates after ankle arthrodesis. The fixation model with two crossed screws and anterior plates and the fixation model with three screws were superior to the fixation model with anterior plates and the fixation model with anterior plates and posterior–lateral screws with respect to resistance to intorsion and extorsion. The fixation model with two crossed screws and anterior plates, and the fixation model with anterior plates and posterior–lateral screws were superior to the fixation model with three screws and the fixation model with anterior plates with respect to resistance to neutral (vertical) stress and dorsiflexion intorsion.

## Conclusion

A comprehensive analysis of the stability and safety of the four models revealed that the fixation model with two crossed screws and anterior plates had better biomechanical performance among the four fixation models, it had the smallest maximum displacement under dorsiflexion torsion, extorsion, intorsion, and neutral vertical load compared to the other three fixation models. with respect to dorsiflexion and intorsion torque, the fixation model with two crossed screws and anterior plates had the best safety among the four models.

Based on the findings of this study, basic anterior plate fixation with three screws has a weak compression effect, is not very safe, and is best reserved for patients with healthy bones. The combination application of a plate and screws provides superior biomechanical effects and greater fixation strength compared to either plate or screw fixation alone. For patients requiring greater fixation strength, the combined application of plate screw is a better choice. The small anterior plate can counter the strength of the Achilles tendon, and the reduction of its size can reduce local blood flow and soft tissue injury in patients, providing an alternative surgical option for patients with poor local soft tissue conditions in the operative area.

However, this study had many shortcomings: ① It failed to consider the influence of soft tissues, such as ligaments and muscles, on ankle joints and ② most of the patients undergoing ankle arthrodesis are middle-aged and elderly patients with osteoporosis, and their bone conditions are closely related to the loosening of screws. Our analysis is limited to use of the software alone, and additional biomechanical experiments need to be performed to verify the findings.

## Data Availability

The data sets used and/or analysed during the current study available from the corresponding author on reasonable request.
